# Supplementary material for: A fault-intrusion-tolerant system and deadline-aware algorithm for scheduling scientific workflow in the cloud
Source: PeerJ Comput Sci. 2021 Nov 2;7:e747. doi: 10.7717/peerj-cs.747 (PMC8576554; doi:10.7717/peerj-cs.747)
Supplement: Supplemental Information 6 [file peerj-cs-07-747-s006.zip › Myproject1.1/docs/org/cloudbus/cloudsim/lists/CloudletList.html]

CloudletList


JavaScript is disabled on your browser.


- Overview
- Package
- Class
- Use
- Tree
- Deprecated
- Index
- Help

- Prev Class
- Next Class

- Frames
- No Frames

- All Classes

- Summary:
- Nested |
- Field |
- Constr |
- Method

- Detail:
- Field |
- Constr |
- Method


org.cloudbus.cloudsim.lists

## Class CloudletList

- java.lang.Object
- - org.cloudbus.cloudsim.lists.CloudletList

- ---

    

  ```
  public class CloudletList
  extends java.lang.Object
  ```

  CloudletList is a collection of operations on lists of Cloudlets.

  Since:
  :   CloudSim Toolkit 2.0

- - ### Constructor Summary

    Constructors

    Constructor and Description

    `CloudletList()`
  - ### Method Summary

    Methods

    | Modifier and Type | Method and Description |
    | --- | --- |
    | `static <T extends Cloudlet>  T` | `getById(java.util.List<T> cloudletList, int id)` Gets the by id. |
    | `static <T extends Cloudlet>  int` | `getPositionById(java.util.List<T> cloudletList, int id)` Returns the position of the cloudlet with that id, if it exists. |
    | `static <T extends Cloudlet>  void` | `sort(java.util.List<T> cloudletList)` Sorts the Cloudlets in a list based on their lengths. |

    - ### Methods inherited from class java.lang.Object

      `clone, equals, finalize, getClass, hashCode, notify, notifyAll, toString, wait, wait, wait`

- - ### Constructor Detail


    - #### CloudletList

      ```
      public CloudletList()
      ```
  - ### Method Detail


    - #### getById

      ```
      public static <T extends Cloudlet> T getById(java.util.List<T> cloudletList,
                                   int id)
      ```

      Gets the by id.

      Parameters:
      :   `cloudletList` - the cloudlet list
      :   `id` - the id

      Returns:
      :   the by id


    - #### getPositionById

      ```
      public static <T extends Cloudlet> int getPositionById(java.util.List<T> cloudletList,
                                             int id)
      ```

      Returns the position of the cloudlet with that id, if it exists. Otherwise -1.

      Parameters:
      :   `cloudletList` - - the list of cloudlets.
      :   `id` - - the id we search for.

      Returns:
      :   - the position of the cloudlet with that id, or -1 otherwise.


    - #### sort

      ```
      public static <T extends Cloudlet> void sort(java.util.List<T> cloudletList)
      ```

      Sorts the Cloudlets in a list based on their lengths.

      Parameters:
      :   `cloudletList` - the cloudlet list


- Overview
- Package
- Class
- Use
- Tree
- Deprecated
- Index
- Help

- Prev Class
- Next Class

- Frames
- No Frames

- All Classes

- Summary:
- Nested |
- Field |
- Constr |
- Method

- Detail:
- Field |
- Constr |
- Method
